# Medical College of the State of South Carolina

**Published:** 1841-08

**Authors:** 


### MEDICAL COLLEGE OF THE STATE OF SOUTH CAROLINA.

The very respectable Professor Dickson, has favoured us with a copy of the annual announcement of the college, with his valedictory address to the class. The number of students in the late session, was nearly 150; of whom 41 were from seven other states of the South, chiefly North Carolina and Georgia, all the rest from South Carolina. The number of graduates was 51, of which 41 were from the state. The last class of the Medical Institute of Louisville, consisted of 208, but 71 of whom were of the state of Kentucky, the remaining 137 from 14 other states. More than two-thirds of the whole class in the former, were of Carolina, while only about one third in the latter, were of Kentucky, a point of view, under which the two schools differ in a very remarkable degree. In every thing, the palmetto state seems to be exclusive. There is another difference in the statistics of these institutions. The former graduated 51 out of less than 150—the latter but 48, of 208. From this we infer that a larger proportion of the students of the South, continue their university studies till graduation, than of the West.

But we must leave these statistics and the train of reflections which they start, to say a word of the Professor's valedictory. Many of these addresses, including our own, have in them scarcely any solid substance; but no complaint of this kind can be made of that before us. With very few exceptions, it abounds in most wholesome and pertinent advice, uttered in a style of great purity and polish. We were particularly struck with the elevation of its moral and religious views. The author's exhortation to "mildness, gentleness, and courtesy, mingled with a dignified self-respect," and his admonition against carrying or using concealed weapons, are worthy of a guardian of young men, destined to labour in the very bosom of society, at a most honorable vocation. But why should he have passed by duelling? It is impossible for us to believe, that the author of an address which breathes such a pure and peaceful spirit, can approve of that savage custom; or that a man of his high intellectual endowments can admit its necessity to the well being of society. D.

